# Seed Priming with Endophytic *Bacillus subtilis* Modulates Physiological Responses of Two Different *Triticum aestivum* L. Cultivars under Drought Stress

**DOI:** 10.3390/plants9121810

**Published:** 2020-12-21

**Authors:** Oksana Lastochkina, Darya Garshina, Sergey Ivanov, Ruslan Yuldashev, Regina Khafizova, Chulpan Allagulova, Kristina Fedorova, Azamat Avalbaev, Dilara Maslennikova, Massimo Bosacchi

**Affiliations:** 1 Institute of Biochemistry and Genetics—Subdivision of the Ufa Federal Research Centre of the Russian Academy of Sciences, Ufa 450054, Russia; yuldashevra@gmail.com (R.Y.); allagulova-chulpan@rambler.ru (C.A.); kristina-iva@yandex.ru (K.F.); avalbaev@yahoo.com (A.A.); dishaoil@mail.ru (D.M.); 2 Bashkir Research Institute of Agriculture—Subdivision of the Ufa Federal Research Centre of the Russian Academy of Sciences, Ufa 450059, Russia; dariya.greatfire@mail.ru; 3 Ufa Institute of Chemistry—Subdivision of the Ufa Federal Research Centre of the Russian Academy of Sciences, Ufa 450054, Russia; ivanov_sp@anrb.ru (S.I.); khafizova_rr@mail.ru (R.K.); 4 KWS Gateway Research Center, Saint Louis, MO 63132, USA; Massimo.Bosacchi@kws.com

**Keywords:** PGPB, endophytic *Bacillus subtilis*, *Triticum aestivum* L., priming, drought tolerance, photosynthetic pigments, oxidative and osmotic status, water holding capacity, salicylic acid

## Abstract

The protective effects against drought stress of the endophytic bacterium *Bacillus subtilis* 10-4 were measured by studying the priming response in two wheat (*Triticum aestivum* L.)—Ekada70 (E70) and Salavat Yulaev (SY)—lines, tolerant and susceptible to drought, respectively. *B. subtilis* 10-4 improved germination and growth parameters under normal conditions in both cultivars with the most pronounced effect observed in cv. E70. Under drought conditions, *B. subtilis* 10-4 significantly ameliorated the negative impact of stress on germination and growth of cv. E70, but had no protective effect on cv. SY. *B. subtilis* 10-4 induced an increase in the levels of photosynthetic chlorophyll (Chl) a, Chl b, and carotenoids (Car) in the leaves of cv. E70, both under normal and drought conditions. In cv. SY plants, bacterial inoculation decreased the contents of Chl a, Chl b, and Car under normal conditions, but pigment content were almost recovered under drought stress. *B. subtilis* 10-4 increased water holding capacity (WHC) of cv. E70 (but did not affect this parameter in cv. SY) and prevented the stress-induced decline in WHC in both cultivars. Notably, *B. subtilis* 10-4 increased endogenous salicylic acid (SA) concentration in both cultivars, especially in cv. E70. Moreover, *B. subtilis* 10-4 reduced drought-induced endogenous SA accumulation, which was correlated with the influence of endophyte on growth, indicating a possible involvement of endogenous SA in the implementation of *B. subtilis*-mediated effects in both cultivars. Overall, *B. subtilis* 10-4 inoculation was found to increase drought tolerance in seedlings of both cultivars, as evidenced by decreased lipid peroxidation, proline content, and electrolyte leakage from tissues of wheat seedlings primed with *B. subtilis* 10-4 under drought conditions.

## 1. Introduction

*Triticum aestivum* L. (wheat) is one of the most widespread valuable cereal food crops [1]. Abiotic stresses leading to drought significantly reduce wheat growth and yield [1,2,3]. Drought affects all elements of plant metabolism by limiting germination, photosynthesis, water and nutrition uptake, increasing transpiration, respiration, oxidative, and osmotic damage to membranes with the cumulative effect of plant growth inhibition [4,5]. Seedling germination and growth in the early stages of ontogenesis are critical stages that predict subsequent plant development and agricultural yield [6]. Priming of seeds (pre-sowing treatment) with beneficial plant growth-promoting bacterial (PGPB) inoculums is an attractive biotic strategy to improve germination rates under adverse environmental conditions and to activate plant defense mechanisms at early stages of plant development [6,7,8,9]. Plants subjected to primary restriction trigger a set of temporary metabolic adaptations that serve as an imprinting mechanism, allowing them to more effectively adapt to subsequent stresses [6]. At present, positive effects of PGPB on plant growth and development under different abiotic stresses (drought, extreme temperatures, salinity, UV radiation, etc.) have been observed in numerous plants including wheat [1,2,7,10,11]. Among PGPB, special attention is given to endophytic bacteria, in particular *Bacillus subtilis*, which colonizes the internal host tissues and positively influence plant metabolism from inside during the entire process of ontogenesis [1,12]. The underlying mechanisms of interaction between endophytic bacteria and host plants under abiotic stresses are mainly unknown. According to present knowledge, the growth-stimulating and anti-stress effects of PGPB including *B. subtilis* on plants are based on multiple mechanisms [1,8,9,10]. The beneficial plant-endophyte associations may improve plant growth and development through direct and indirect mechanisms: by enhancing mineral uptake, diluting pathogen access through biological competition, producing a variety of bioactive compounds (auxins, biosurfactants, siderophores, enzymes, etc.), and regulating the level of some important plant hormones including salicylic acid (SA), which plays a key role in augmenting tolerance response under abiotic stresses [1,13,14,15,16,17]. However, it is still far from clear how endophytic bacteria regulate the defense systems of host plants and increase plant stress tolerance to drought. Moreover, the efficiency of the same bacterial strain may vary depending on a number of factors including the type of host plant, its varietal characteristics, ecological/geographical origin, etc. [1,11,18,19,20]. As a result of natural and artificial selection under varying types of drought, wheat plants growing in different geographical/ecological regions formed groups with different drought adaptation strategies [21,22,23,24]. It is of great interest to study the spectrum of protective mechanisms involved in the formation of *B. subtilis*–induced drought tolerance of wheat plants with contrasting ability to cope with drought stress and having evolutionary formed different drought adaptation strategies.

This study evaluated the effect of seed priming with *B. subtilis* 10-4, under normal and drought stress conditions, on growth and tolerance (some morpho-physiological and biochemical parameters) of wheat plants with contrasting behavior under drought in early stages of ontogenesis.

## 2. Materials and Methods

### 2.1. Bacterial Strain and Inoculum Preparation

The bacterium *Bacillus subtilis* (strain 10-4) was isolated from the arable soils at the Bashkir Research Institute of Agriculture of the Ufa Federal Research Center of the Russian Academy of Sciences (BRIA UFRC RAS) (Ufa, Russia), identified using 16S rRNA and characterized [11]. *B. subtilis* 10-4 cells were cultured in potato glucose agar (PGA) medium at 37 °C for 3–4 days [11,25]. To obtain the initial inoculum of *B. subtilis* 10-4, a suspension culture was grown to a concentration of 10^8^ CFU mL^−1^, according to the 0.5 McFarland Turbidity Standard, monitored at an optical density of 600 nm (OD600) (SmartSpec^TM^ Plus spectrophotometer, Bio–Rad, Hercules, CA, USA), and then diluted down to 10^5^ CFU mL^−1^ using distilled water [11].

### 2.2. Plant Materials, Growth Conditions, and Growth Parameters Assay

Spring wheat seeds (*Triticum aestivum* L., drought-sensitive cv. Salavat Yulaev (SY) and drought-tolerant cv. Ekada70 (E70)) were obtained from the Chishminsky Breeding Station of the BRIA UFRC RAS (Chishmy, Bashkortostan, Russia). The experiments were carried out under laboratory conditions using hydroponically cultured seedlings [26]. The seeds were sterilized in 97% ethyl alcohol for 60 s, washed five times with tap water, and then immersed into solutions of *B. subtilis* 10-4 (10^5^ CFU mL^−1^) [11] or water (control) for 1 h. Bacterial primed (test) and non-primed (control) seeds were grown on filter paper with tap water for three days under a long-day photoperiod (16 h light/8 h dark, 22–23 °C). Thereafter, three day old seedlings were transferred to glasses with water (control) or 12% PEG–6000 (drought) and grown further in the same conditions. Plant samples were taken after 3, 7, 24, 48, and 72 h to assess the physio-biochemical parameters (each variant was carried out in three biological replicates with 30 seedlings per replicate). Changes in the length of wheat roots and shoots and their biomass accumulation (fresh (FW) and dry (DW) weights) were determined in six day old seedlings according to [26]. Each variant was carried out in three replicates with 100 seedlings per replicate.

### 2.3. Seed Germination Assay

To assess the drought tolerance of seeds during germination, we measured the ability of seeds to germinate in solutions of sucrose (16, 18, 20%, resulting in osmotic potentials of 1.32, 1.44, and 1.56 MPa, respectively) and PEG–6000 (3, 6, 9, 12%, resulting in osmotic potentials of −0.11, −0.23, and −0.45 MPa, respectively) [27]. The bacterial-primed and non-primed seeds were sown on Petri dishes with filter paper soaked with 5 mL of sucrose solutions and/or PEG–6000 (tests) and water (control) (100 seeds per dish, three replicates). The seeds were grown for five days in the dark at 21 °C, after which the number of germinated seeds was counted. The percentage of germination was determined by the number of seeds giving a rootlet of the smallest length [26].

### 2.4. Seedlings Colonization Assay

Colonization of wheat seedlings by bacteria was assayed using surface-sterilized three-day old seedlings primed with *B. subtilis* 10-4 (10^5^ CFU ml^−1^) and grown under sterile conditions [11]. Wheat leaf and root segments were sterilized in 70% ethyl alcohol for 5 min, washed with sterile water (three times), and kept at 30 °C for 24 h in Petri dishes on a gel matrix made with *Bacillus* ChromoSelect Agar (Sigma Aldrich, St. Louis, MO, USA). Random amplification of polymorphic DNA—polymerase chain reaction (RAPD–PCR) analysis using AFK primers (5′-GCGTCCATTC-3′) was used to assess the identity of bacteria grown around plant segments with origin strain 10-4 [11].

### 2.5. Determination of Photosynthetic Pigments

Photosynthetic pigments, chlorophyll (Chl) a, Chl b, and carotenoids (Car) were assayed as described [26,28]. Plant leaves (0.05 g) were homogenized in 90% ethanol (10 mL) with the addition of CaCO_3_ and filtered. The optical density of the filtered extracts was measured using a SmartSpecTM Plus spectrophotometer (Bio–Rad, Hercules, CA, USA) at 663 (Chl a), 646 (Chl b), and 470 nm (Car).

### 2.6. Determination of Water Holding Capacity (WHC) of Leaves

The WHC of leaves was assayed as described [29]. In summary, three fresh leaves of 21-day old seedlings were cut, weighed, and maintained for 3–4 h under condition of 50% RH and 25 °C. The desiccated leaves were weighed and then dried at 105 °C. WHC was determined as a percentage of the total water content [29].

### 2.7. Assessments of Proline (Pro), Electrolyte Leakage (EL), and Lipid Peroxidation (MDA)

Pro content was evaluated as described [30] with the modification [31]. Disturbance of the barrier properties of cell membranes was assayed by following electrolyte leakage (EL) from plant tissues using an OK 102/1 conductometer (Radelkis, Hungary), measuring the ohmic resistance of water extracts on constant current [32]. The lipid peroxidation was assessed by malondialdehyde (MDA) content [26]. The optical densities were measured using a SmartSpecTM Plus spectrophotometer (Bio–Rad, Hercules, CA, USA) at 522 nm (for Pro) and 532 nm and 600 nm (for MDA).

### 2.8. Endogenous Salicylic Acid (SA) Extraction and Quantification

Endogenous SA was assayed by the high-performance liquid chromatography (HPLC) method [33]. In summary, plant tissues (0.2–0.3 g) were extracted with 20 mL of distilled water (90–100 °C), incubated at 100 °C for 30 min, and cooled. The obtained extracts were filtered through a membrane filter (0.45 µm) (Chromafil Xtra PTFE–45/13, Macherey-Nagel GmbH Co, Duren, Germany). The analysis was performed using a Waters Breeze chromatography (Waters, Milford, MA, USA) with a SPD M20A diode array detector at 305 nm. A 250 × 4.6 mm Pursuit C18, 5 μm column (Agilent Technologies, Santa Clara, CA, USA) was used. As the mobile phase, an eluent of the composition of the 0.5% solution of H_3_PO_4_:acetonitrile = 65:35 (1.0 mL/min) was used. A total of 20 μL of the analyzed solutions (extracts) were introduced into the chromatographic system using a Waters 2707 autosampler (Waters, Milford, MA, USA). The software calibration curve was used for the total SA content calculation.

### 2.9. Statistical Analysis

All microbiological, molecular, physiological, and biochemical experiments were performed in three biological replicates. This study used three biological replicates with 100 seeds/seedlings per replicate to assess seed germination and wheat growth parameters. To assess the physio-biochemical parameters, we performed three biological replicates with 30 seedlings per replicate. The data were presented as the mean ± standard error (±SEM). Statistically significant differences between the mean values were evaluated by two-way analysis of variance (ANOVA), followed by the Tukey test (*p* < 0.05).

## 3. Results

### 3.1. Seedling Colonization with Bacillus subtilis 10-4

**Figure 1:**
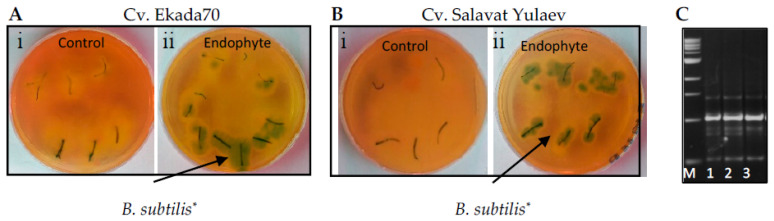
The ability of *Bacillus subtilis* 10-4 (endophyte) to colonize internal tissues of surface-sterilized wheat plants. Two cultivars were tested: cv. Ekada70 (drought-tolerant)) (**A**) and cv. Salavat Yulaev (drought-sensitive) (**B**). **i**—the absence of *B. subtilis* bacteria growth around the surface-sterilized root and leaf segments in non-inoculated wheat seedlings (Control); **ii**—*B. subtilis* bacterial growth (* light green to green colonies) around the surface-sterilized leaf and root segments of wheat seedlings pre-inoculated with *B. subtilis* 10-4 (Endophyte); (**C**)—electrophoregram of PAAG: M—DNA marker; 1—DNA of origin *B. subtilis* 10-4, used for pre-sowing inoculation of seeds; 2 and 3—DNA of bacteria grown around wheat plant segments. Black arrows indicate *B. subtilis* growth (light green to green colonies) around the plant segments. Each variant was performed in three replicates, 10 seedlings per replicate.

RAPD–PCR analysis using surface-sterilized three day old wheat seedlings demonstrated the ability of *B. subtilis* 10-4 to colonize internal tissues of drought-tolerant cv. E70 and drought-sensitive cv. SY wheat plants (Figure 1A,B). Bacterial growth was absent from non-inoculated wheat plant segments (Figure 1(Ai,Bi)) and present in segments pre-inoculated with *B. subtilis* 10-4 (Figure 1(Aii,Bii)). RAPD–PCR analysis was used to correlate the identities of the *B. subtilis* 10-4 inoculum and resulting growth (Figure 1C).

### 3.2. Germination

**Figure 2:**
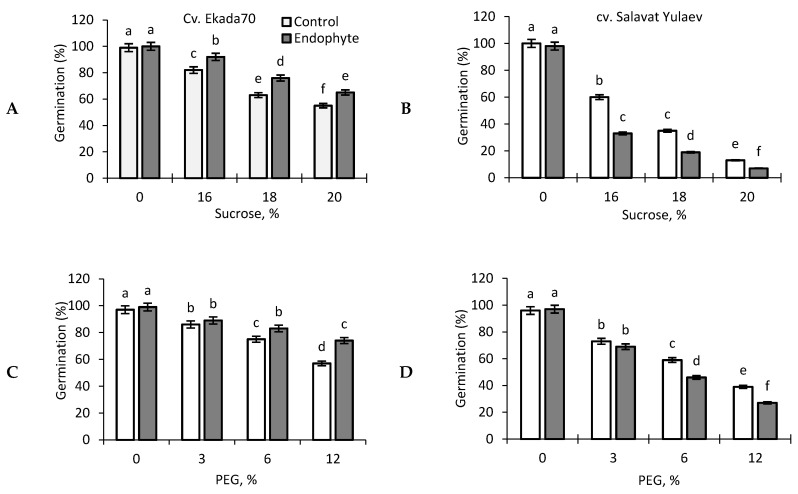
Effect of endophytic *B. subtilis* 10-4 (Endophyte) on the percentage of seed germination of wheat cv. Ekada70 (**A**,**C**), and cv. Salavat Yulaev (**B**,**D**) under high gradients of sucrose (Sucrose) (0, 16%, 18%, 20%) (**A**,**B**) and PEG–6000 (PEG) (0, 3%, 6%, 12%) (**C**,**D**). Control—non-inoculated seeds; Endophyte—seeds pre-inoculated with *B. subtilis* 10-4. The bars are the means of three repetitions ± SEM (n = 100, three replicates). Different letters indicate a significant difference between the means at the probability level of *p* < 0.05.

In vitro germination experiments showed that seeds of wheat cv. Ekada70 (E70) (drought–tolerant) have better germination percentages than cv. Salavat Yulaev (SY) (drought-susceptible) on high sucrose and PEG–6000 gradients (Figure 2A–D). *B. subtilis* 10-4 ameliorated the negative impact of drought (caused by high gradients of sucrose and PEG–6000) on seed germination of cv. E70 (Figure 2A,C), but exacerbated these effects under the same conditions in the case of cv. SY (Figure 2B,D). Interestingly, under normal growth conditions, the percentage of seed germination pre-inoculated with *B. subtilis* 10-4 was higher for cv. E70 (Figure 2A,B), but not for cv. SY, with no significant differences from non-inoculated control ones (Figure 2B,D).

### 3.3. Seedling Growth

**Figure 3:**
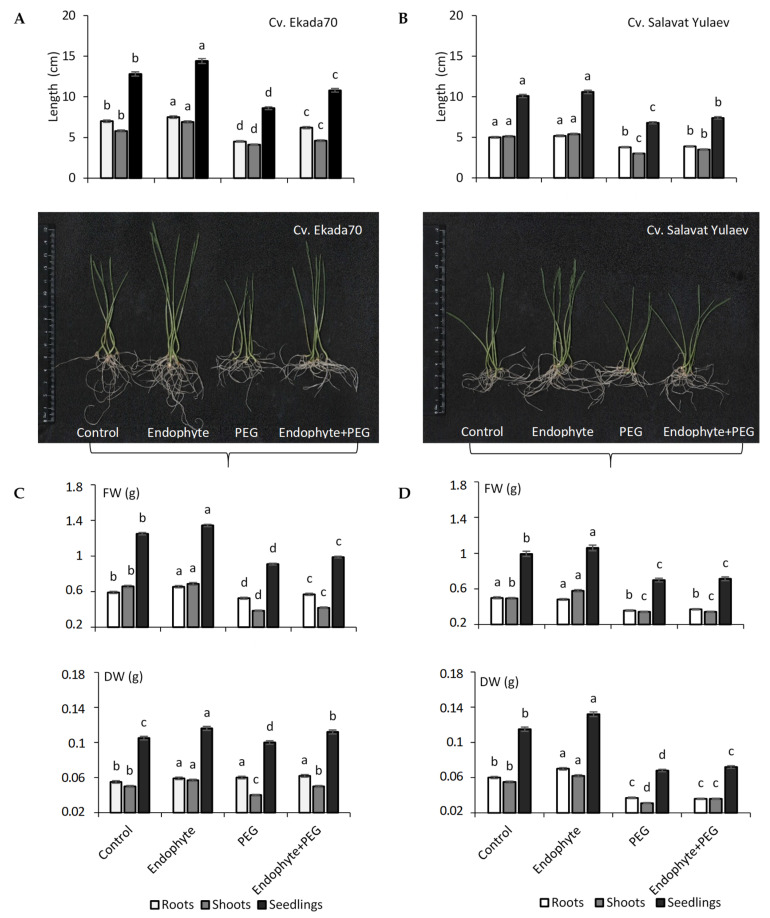
Effect of endophytic *B. subtilis* 10-4 (endophyte) on the elongation of six-day old seedlings (length of the roots, shoots, and their visual appearance), fresh (FW), and dry biomass (DW) accumulation of wheat of cv. Ekada70 (**A**,**C**) and cv. Salavat Yulaev (**B**,**D**) under normal and drought (12% PEG–6000) conditions. The bars are the means of three repetitions ± SEM (n = 100, three replicates). FW, fresh weight; DW, dry weight. Different letters indicate a significant difference between the means at the probability level of *p* < 0.05.

Under normal growth conditions, inoculation with *B. subtilis* 10-4 prior to sowing resulted in increased root and shoot length in six-day old wheat seedlings, with the most pronounced effect observed in cv. E70 (Figure 3A). In particular, endophyte application increased (up to 20–30%) the length of wheat seedlings (roots and shoots) of cv. E70 (Figure 3A), compared to increases of 10% or lower relative to the control, as observed in cv. SY (Figure 3B). A similar picture under normal growth conditions was observed with regard to the seedlings’ fresh (FW) and dry (DW) biomass accumulation in the early stages of wheat ontogenesis (Figure 3C,D).

Exposure to drought resulted in about a 1.5–2 fold decrease in wheat root and shoot length in both studied cultivars (Figure 3A,B). Priming with endophyte *B. subtilis* 10-4 significantly mitigated (by 1.3–1.5 times) the degree of drought-induced damage on the growth processes of cv. E70 (Figure 3A). However, for cv. SY, there was no detectable protective effect on seedling growth at six days (Figure 3B). Cv. E70 seedlings had longer root and shoot lengths compared to non-inoculated control plants under the same drought conditions (Figure 3A). Similar effects of *B. subtilis* 10-4 were observed on fresh (FW) and dry (DW) biomass accumulation in plants of both cultivars (Figure 3C,D). Drought significantly decreased FW and DW in both cultivars (Figure 3C,D). However, inoculation with endophyte *B. subtilis* 10-4 reduced the negative impact of drought on FW and DW for both cv. E70 (up to 30%) (Figure 3C) and cv. SY (Figure 3D). Thus, it was discovered that the application of endophyte *B. subtilis* 10-4 conferred effective protection to drought-tolerant cv. E70 plants (Figure 3A,C). In contrast, drought-sensitive cv. SY plants did not show the same protective responses on plant growth following *B. subtilis* 10-4 inoculation under drought conditions (Figure 3B,D).

### 3.4. Photosynthetic Pigments

**Figure 4:**
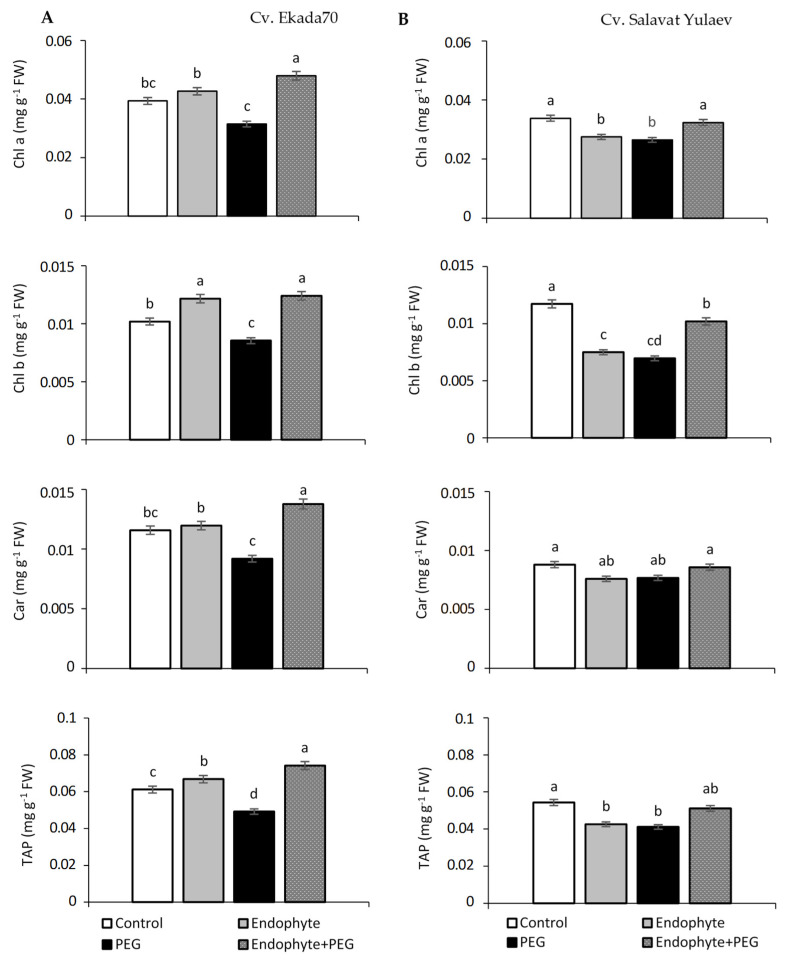
Effect of endophytic *B. subtilis* 10-4 (endophyte) on photosynthetic pigments in the leaves of wheat seedlings of cv. Ekada70 (**A**) and cv. Salavat Yulaev (**B**) under normal and drought stress conditions. Chl a—chlorophyll a; Chl b—chlorophyll b; Car—carotenoids; TAP—total amount of pigments. (n = 30, three replicates). Different letters indicate a significant difference between the means at the probability level of *p* < 0.05. Significant difference compared with the control (*p* < 0.05).

Our results showed that *B. subtilis* 10-4 influences photosynthetic pigment accumulation differently in wheat leaves of cv. E70 and cv. SY under both normal and drought conditions (Figure 4). In cv. E70 leaves under normal growth conditions, *B. subtilis* 10-4 promoted a slight increase in chlorophyll (Chl) a (up to 1.1 times), Chl b (up to 1.2 times), and carotenoid (Car) (up to 1.09 times) content, with the total amount of photosynthetic pigments (TAP) exhibiting a 1.12-fold increase (Figure 4A). In contrast, wheat leaves of cv. SY exhibited a reduction in in the photosynthetic pigments of l.2 (Chl a), 1.6 (Chl b), 1.16 (Car), and 1.29 (TAP)-fold (Figure 4B).

Drought exposure over 24 h resulted in a 2–3 fold decrease in the content of Chl a, Chl b, and Car in wheat leaves of both cultivars (Figure 4A,B). At the same time, wheat leaves primed with *B. subtilis* 10-4 exhibited increased levels of the assessed photosynthetic pigments with the most pronounced effect for cv. E70, whereas the contents of Chl a, Chl b, Car, and TAP increased up to 1.52, 1.46, 1.52, 1.51 times, respectively (Figure 4A). Moreover, for cv. E70, the content of these pigments in endophyte-inoculated plants were even higher than in inoculated plants grown under normal conditions (Figure 4A). Endophyte inoculation also increased photosynthetic pigment content in cv. SY plants under drought stress in comparison with non-inoculated ones, but did not exceed the values of non–inoculated control plants grown under normal conditions (Figure 4B). With that, it should be noted that wheat leaves of cv. E70 contained a significantly higher total amount of all studied pigments (Figure 4A). Thus, drought resulted in a 2–3-fold decrease in Chl a, Chl b, and Car contents in leaves of both wheat cultivars, however, *B. subtilis* 10-4 significantly increased these photosynthetic pigments cv. E70 in contrast with cv. SY, where the observed increase did not exceed that of control plants grown under normal conditions.

### 3.5. Water Holding Capacity (WHC)

**Figure 5:**
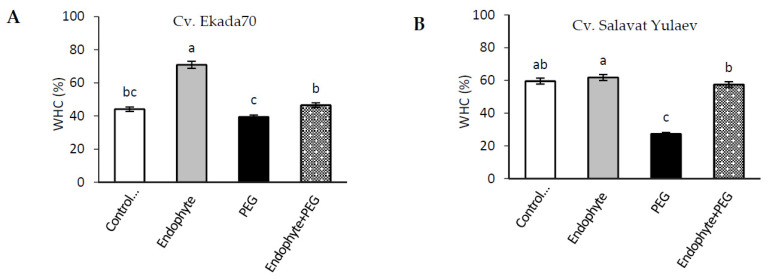
Effect of endophytic *B. subtilis* 10-4 (endophyte) on the water-holding capacity (WHC) in leaves of wheat seedlings of cv. Ekada70 (**A**) and cv. Salavat Yulaev (**B**) under normal and drought conditions (21 days after sowing of seeds). C—non-primed plants grown under normal conditions; Endophyte—plants primed with *B. subtilis* 10-4 and grown under normal conditions; PEG—non-primed plants are grown under drought (12% PEG-6000); Endophyte + PEG—plants primed with *B. subtilis* 10-4 and grown under drought (12% PEG-6000). The bars are the means of three repetitions ± SEM. Different letters indicate a significant difference between the means at the probability level of *p* < 0.05.

This study revealed that under normal growth conditions, WHC of wheat leaves in the tillering phase (21-day old seedlings) differed between cultivars from 44.1% (drought-tolerant cv. E70) (Figure 5A) to 59.5% (drought-sensitive cv. SY) (Figure 5B). Under normal growth conditions, pre–inoculation with *B. subtilis* 10-4 led to an increase in WHC in both cultivars ranging from 70.9% (cv. E70) (Figure 5A) to 61.7% (cv. SY) (Figure 5B). In cv. SY, the values of the WHC of endophyte-inoculated plants (61.7%) were comparable with those of non–inoculated control plants (59.5%). However, endophyte inoculation resulted in a significant increase in the WHC of leaves of cv. E70 (from 44.1% to 70.9%), indicating that this drought-tolerant cultivar is more responsive to *B. subtilis* 10-4 inoculation. Drought stress triggered a 2-fold decrease in the WHC of leaves of drought-sensitive cv. SY (Figure 5B) and had practically no effect on the WHC of drought-tolerant cv. E70 (Figure 5A). Inoculation with *B. subtilis* 10-4 prevented drought-induced decline in the WHC of cv. SY where the amplitude of changes was dramatically pronounced due to the stronger injurious effect of drought (Figure 5B). As a result, in both cultivars, E70 and SY, pre-inoculated with *B. subtilis* 10-4, the WHC indices under drought were comparable with non-inoculated control plants grown under normal conditions (Figure 5A,B).

### 3.6. Electrolyte Leakage and Lipid Peroxidation

**Figure 6:**
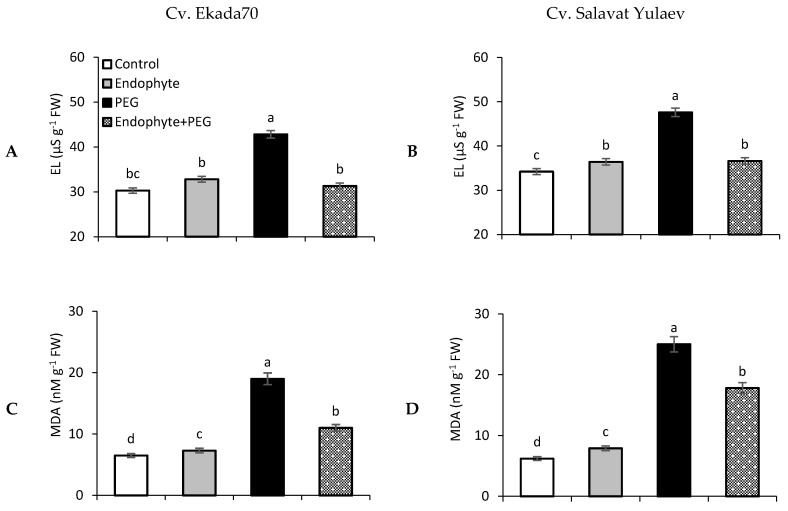
Changes in electrolyte leakage (EL) from six-day old seedling tissues and lipid peroxidation (malondialdehyde (MDA)) of cv. Ekada70 (**A**,**C**) and cv. Salavat Yulaev (**B**,**D**) primed before sowing with endophytic *B. subtilis* 10-4 (endophyte) and grown under normal and drought (24 h exposure to 12% PEG–6000) conditions. Control—non-primed plants grown under normal conditions; Endophyte—plants primed with *B. subtilis* 10-4 and grown under normal conditions; PEG—non-primed plants are grown under drought (12% PEG–6000); Endophyte + PEG—plants primed with *B. subtilis* 10-4 and grown under drought (12% PEG–6000). The bars are the means of three repetitions ± SEM. Different letters indicate a significant difference between the means at the probability level of *p* < 0.05.

Exposure to drought over 24 h resulted in strong damage to plant cells as measured by an approximately 2-fold increase in the intensity of electrolyte leakage (EL) from plant tissues (six-day old seedlings) of both studied wheat cultivars E70 and SY in comparison to the control (Figure 6A,B). Priming with *B. subtilis* 10-4 led to a much lower level of drought–activated EL, thereby demonstrating the efficiency of bacterial inoculation of the plants for their protection against drought, with the most pronounced protective effect for cv. E70 (Figure 6A). With that, the level of EL was also lower for cv. E70 both under normal and drought conditions (Figure 6A) in comparison with the same parameters for cv. SY (Figure 6B). No significant changes were observed in this indicator of cell membrane integrity after *B. subtilis* 10-4 priming of both cultivars of E70 and SY plants under normal conditions (Figure 6A,B).

A similar pattern was observed for changes in MDA content in wheat seedlings of both studied cultivars primed with *B. subtilis* 10-4 and exposed to drought (Figure 6C,D). Drought significantly increased (up to approximately 3-fold) the MDA content in wheat seedlings, especially for cv. SY (Figure 6C). Priming with endophyte *B. subtilis* 10-4 decreased (by approximately 2-fold) such drought–caused MDA generation in wheat seedlings of both cultivars E70 and SY. However, the protective effect was more pronounced in cv. E70 plants (Figure 6C). There was an insignificant difference in the MDA content of endophyte–primed and non–primed wheat seedlings of both cultivars, E70 and SY, under normal conditions (Figure 6C,D).

### 3.7. Proline (Pro)

**Figure 7:**
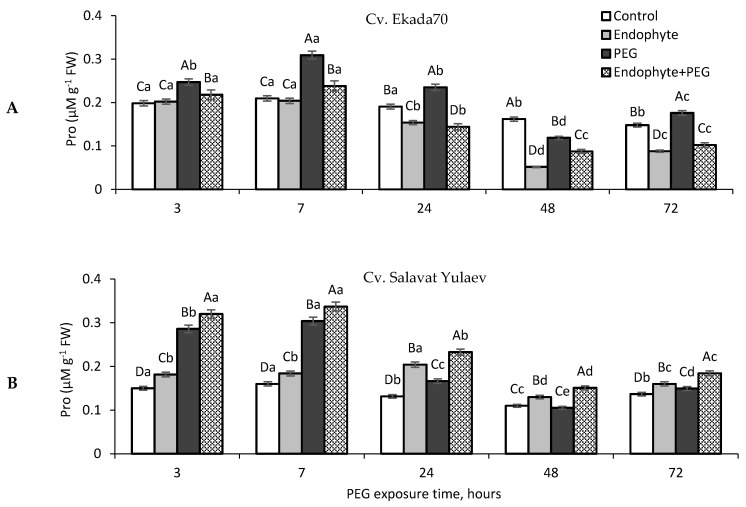
Changes in Proline (Pro) concentration in wheat seedlings (six days old) of cv. Ekada70 (drought-tolerant) (**A**) and cv. Salavat Yulaev (drought-sensitive) (**B**) primed with endophytic *B. subtilis* 10-4 (endophyte) and grown under normal and drought conditions (time of 12% PEG-6000 exposure are 3, 7, 24, 48, 72 h). Control—non-primed wheat seedlings grown under normal conditions; Endophyte—wheat seedlings primed with *B. subtilis* 10-4 and grown under normal conditions; PEG—non-primed wheat seedlings grown under drought (12% PEG-6000); Endophyte + PEG—wheat seedlings primed with *B. subtilis* 10-4 and grown under drought (12% PEG-6000). The bars are the means of three repetitions ± SEM. Different lowercase letters on top of the columns indicate that means for each treatment at different time points are different at *p* < 0.05. Different capital letters on top of the columns indicate that means for the same time point of various treatments are different at *p* < 0.05.

Drought related treatments resulted in transient Pro accumulation in wheat seedlings of cv. E70, with a peak of 140% compared to the control observed 7 h after stress exposure (Figure 7A). In seedlings of cv. SY, an almost 2-fold accumulation of Pro was observed over 3 h of stress exposure, persisting up to 7 h, then decreasing to the control level after 24 h of stress (Figure 7B). Priming with *B. subtilis* 10-4 led to a decrease in the level of stress-induced Pro accumulation in plants of cv. E70 (Figure 7A), while in the case of cv. SY, a slight additional accumulation of Pro was observed (Figure 7B). Under normal conditions, *B. subtilis* 10-4 caused a small but reliably significant accumulation of Pro in plants of cv. SY (Figure 7B). The same application did not trigger any increase in Pro content in plants of cv. E70, and actually reduced levels relative to the control by the end of the experiment (Figure 7A). Additionally, it should be noted that cv. E70 exhibited an increased Pro content compared to cv. SY (Figure 7A,B).

### 3.8. Endogenous Salicylic Acid (SA)

**Figure 8:**
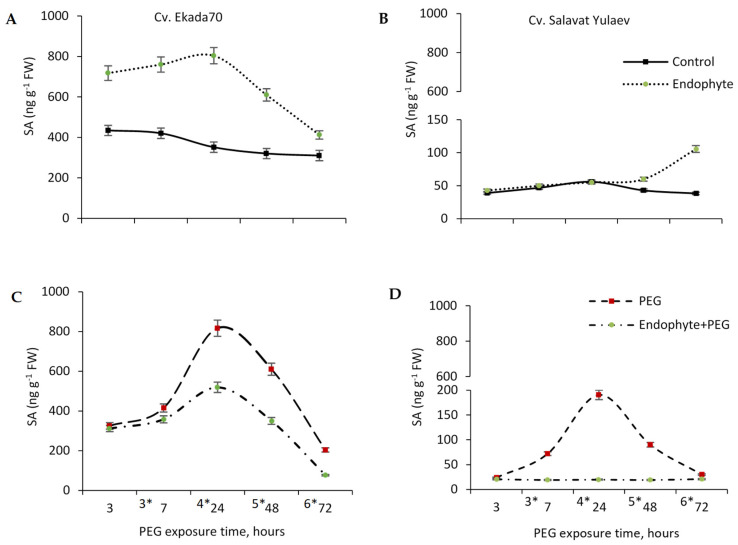
Effect of endophytic *B. subtilis* 10-4 (endophyte) inoculation on endogenous salicylic acid (SA) content in wheat seedlings of cv. Ekada70 (drought-tolerant) (**A**,**C**) and cv. Salavat Yulaev (drought-sensitive) (**B**,**D**) under normal (**A**,**B**) and drought (12% PEG-6000) (PEG) (**C,D**) conditions. The bars are the means of three repetitions ± SEM. Different letters indicate a significant difference between the means at the probability level of *p* < 0.05. FW, fresh weight. 3 *—three days old seedlings, 4 *—four days old seedlings, 5 *—five days old seedlings, 6 *—six days old seedlings.

This study revealed that under normal growth conditions, the content of endogenous SA in wheat plants significantly varied depending on the cultivar (Figure 8). The highest endogenous SA levels were measured in drought-tolerant cv. E70 (285.0–534.1 ng g^−1^ FW) (Figure 8A), and the lowest measured in drought-sensitive cv. SY (38.4–56.1 ng g^−1^ FW) (Figure 8B). Priming with *B. subtilis* 10-4 resulted in increased (up to 1.8–2.5 times) accumulation of endogenous SA in wheat seedlings of cv. E70 and cv. SY, however, the patterns of accumulation were different (Figure 8A,B). In particular, cv. E70 exhibited a fast transient accumulation with a peak at 4-day old seedlings (Figure 8A) while for cv. SY remained static for three days with SA accumulation (up to 2.5 times) becoming observable in 5–6-day old seedlings (Figure 8B).

Under drought stress, a transient increase in endogenous SA was observed, with peaks after 24 h of drought exposure (four days old seedlings) of up to 610.0 ng g^−1^ for cv. E70 and 190.6 ng g^−1^ for cv. SY, gradually decreasing (six day old seedlings, 72 h stress exposure) to 204.2 ng g^−1^ for cv. E70 and 30.0 ng g^−1^ FW for cv. SY (Figure 8C,D). At the same time, priming with *B. subtilis* 10-4 contributed to a decrease in such drought-induced endogenous SA accumulation (up to about 50%) both for cv. E70 (Figure 8C) and cv. SY (Figure 8D).

## 4. Discussion

Priming of seeds with beneficial bacterial inoculums is an attractive ecological approach to improving germination rates under adverse environmental stress conditions, and to activate plant defense mechanisms at the early stages of plant development due to induced tolerance [7,8,9,34,35,36,37,38,39]. Priming affects multiple physiological systems in general, in both seeds and plants and can be defined by the response of primed plants responding to stresses faster and more efficiently [34]. The elucidation of the mechanisms of action of PGPB plays a key role in establishing an effective protocol for their use in minimizing the damaging effects of drought on plants. The manifestation of the physiological effect of PGPB on plants occurs through interrelated direct and indirect mechanisms [1], stimulation of plant growth through improved bioavailability of macro/microelements (nitrogen fixation, phosphate solubilization, iron sequestration), the production of phytohormones and regulation of their level in the host plant, and induction of systemic resistance and tolerance to stresses [1,35,36,37]. Numerous studies have shown that beneficial bacteria could alleviate the adverse effects of drought on the growth and development of varied plants including wheat [1,9,10,16,19,35]. For example, wheat plants inoculated with *Azospirillum brasilense* Sp245 were characterized by an increase in the linear dimensions of the coleoptile, fresh weight, and an improvement in the indicators of the water status of seedlings in comparison with control samples under drought [38]. *A. brasilense* INTA Az-39 increased the yield of wheat grown in dry farming zones due to an increase in the growth rate under the influence of these bacteria, an increase in the level of biomass accumulation, and the number of grains per spikelet [39]. Biopriming with PGPB improved the percentage of seed germination and growth of radish and wheat under salinity [6,11]. Our results showed that priming with endophyte *B. subtilis* 10-4 improved growth in drought-tolerant (DT) cv. E70 (by 30%) and drought-sensitive (DS) cv. SY (by 10%) seedlings, but protective effects on growth under drought stress were observed only for cv. E70 (Figure 2 and Figure 3) in the early stages of ontogenesis. Such differences in wheat responses to combined bacterial priming and drought exposure may be connected with the drought response strategies of these cultivars [19,23]. DS wheat cv. SY is characterized by its slow pace of development and tillering in the early stages of ontogenesis, allowing them to tolerate spring drought well [21]. Application of *B. subtilis* 10-4 in this stage of ontogenesis for cv. SY leads to overlapping developmental programs of plants, which have evolved to contain growth during this phase and exhibit an extended germination rate, a long tillering phase, and rapid root system development. As a result, there is no visible effect on plant growth upon *B. subtilis* 10-4 inoculation under drought or even an inhibitory effect was observed (Figure 2). In contrast, the DT cv. E70 programmed for intensive growth at the beginning of the vegetation period driven by reserved moisture from the spring period in the soil, and during subsequent drought conditions typical of summer, has established a well-branched network of the root system, which contributes to good yield [20,21]. Application of *B. subtilis* 10-4 in this phase of ontogenesis is most likely enhanced by the programmed ability of the plant to grow fast under both normal and drought stress. Understanding the mechanisms behind interactions of endophytic *B. subtilis* 10-4 and how wheat cultivars differ in their drought tolerance adaptive strategies is important to improve the strategies for the use of *B. subtilis* in agriculture.

Photosynthesis is one of the core processes in primary plant metabolism that is directly related to plant biomass productivity and is highly susceptible to drought stress [40,41]. Drought leads to a decrease in both photosynthetic pigment content and photosynthetic efficiency of plants resulting from decreased leaf area, plant height, biomass accumulation, and yield formation. The content of the main photosynthetic pigments chlorophylls (Chl) and carotenoids (Car), while indirect, is the most important biochemical indicator of plant photosynthetic activity [42,43,44]. Many studies have shown that bacterial inoculation may positively influence the maintenance of photosynthetic pigments in plants accompanied by improved drought tolerance [42,45,46]. *B. pumilus* alleviated drought-induced damage to the photosynthetic activity of *Glycyrrhiza uralensis* [42]. In our study of both DT and DS wheat cultivars, a decrease in the Chl a, Chl b, and Car was observed in response to drought stress (Figure 4). *B. subtilis* 10-4 was not only mitigated, but even increased these photosynthetic pigments for DT cv. E70 while in DS cv. SY, it partially diminished the drought-induced decline in the photosynthetic pigment content. This indicated that *B. subtilis* 10-4 more effectively increases the photosynthetic activity of DT wheat cv. E70, while for cv. SY, the positive influence was also observed under drought but was less pronounced than in cv. E70. In general, the findings allowed us to assume that one of the mechanisms of the protective action of bacterial priming on wheat plants under drought is connected with their ability to influence the components of photosynthesis.

Drought causes oxidative stress in plants due to the excessive generation of reactive oxygen species (ROS), which compromises the integrity in the structure and the function membranes, enzyme activity, mutagenesis, and cell cycle, ultimately leading to the death of cells and the whole organism [47,48]. To date, extensive information has been accumulated on the ability of PGPB including *B. subtilis* to trigger the antioxidant system (AOS), which leads to an increase in drought tolerance in the associated host plant [1,9,16,46]. It was revealed that inoculation with *Lactobacillus plantarum* activated catalase activity and increased the integral antioxidant capacity in wheat seedlings [10]. *Bacillus* spp. and *Arthrobacter pascens* increased the activity of antioxidant enzymes (peroxidase, catalase, superoxide dismutase, and ascorbate-peroxidase) in maize and wheat plants [10]. Our results also showed that *B. subtilis* 10-4 significantly decreased drought-induced EL and lipid peroxidation in wheat plants (DT and DS cultivars) (Figure 6), however, the most pronounced protective effect was observed for plants of DT cv. E70. Thus, bacterial priming contributes an important role in plant protection against drought-induced oxidative damages.

Osmotic regulation by PGPB is considered to be another important reaction at the cellular level, helping plants tolerate drought-induced damages [1,49]. It was reported that PGPB produces osmolytes in response to drought stress, which acts synergistically with plant-produced osmolytes and results in plant growth stimulation [50]. Among the different osmolytes (sugars, glycine betaine, organic acids, inorganic ions, etc.) the amino acid proline (Pro) is the most frequent acclimatization response observed in plants and bacteria under water deficiency [9,51], and is thus considered to be an indication of plant drought tolerance [52]. Pro leads to osmotic regulation, scavenging of free radicals, and stabilization of subcellular structures in plant cells to overcome the harmful effects of drought [53]. Several reports have shown that the inoculation of plants with PGPB including *B. subtilis* elevated Pro level in plants under drought, thus increasing growth, biomass accumulation, and leaf water potential during stress [50,54]. Inoculation with *Burkholderia phytofirmans* PsJN increased Pro in grapevine plants under osmotic stress [50]. *Bacillus* spp. enhanced Pro synthesis in plants under water stress, which was accompanied by upregulation of *P5CS* genes as well as inhibiting *ProDH* gene expression, which acts during Pro metabolism [55]. The introduction of *proBA* genes derived from *B. subtilis* into *Arabidopsis thaliana* results in elevated Pro synthesis in transgenic plants and correlated with osmotic tolerance [56]. Endophytic *Sphingomonas* sp. strain LK11 isolated from *Tephrosia apollinea* leaves increased drought tolerance by increasing the production of not only Pro, but also glutamate and glycine in inoculated soybean plants [57,58]. Endophytic *B. amyloliquefaciens* enhanced salt tolerance of *Oryza sativa* plants, an affect that correlated with increased Pro, phenylalanine, cysteine, aspartic, and glutamic acids [59]. In our study, endophytic *B. subtilis* 10-4 prevented drought-induced Pro accumulation in wheat seedlings of DT cv. E70 (Figure 7A), indicating lower levels of drought-induced damage confirmed by the results of the analysis of MDA and EL (Figure 6A). In plants of DS cv. SY, *B. subtilis* 10-4 caused an additional Pro accumulation under drought (Figure 7B). We assumed that in the case of DS wheat cultivar, the detected elevated Pro can play a role in the stabilization of the plants’ AOS under drought and ensure the neutralization of ROS production [53,60], thereby preventing damage to the structures and permeability of cell membranes of cell compartments during drought [53]. In this scenario, different mechanisms of Pro level regulation are implemented under drought, which is effective, as judged by the decrease in the damaging effects of drought on the integrity and permeability of membrane structures (Figure 6), the WHC (which was studied on 21 days of wheat ontogenesis) (Figure 5), and stabilization of photosynthesis (Figure 4), indicating a fairly prolonged protective effect of *B. subtilis* 10-4 on these plants.

Several studies have reported that plants inoculated with PGPB showed improved growth accompanied by increased relative water content (RWC) [1,10]. Wheat plants inoculated with *Azospirillum brasilense* Sp245 under the influence of drought were characterized by a higher level of RWC and water movement along the apoplast in comparison with non-inoculated and stressed plants [38]. The degree of water deficit may be assessed by estimating the index of the water holding capacity (WHC) in plant leaves. The higher WHC of species shows that they are better adapted to environmental stresses such as drought [61]. Our results demonstrated that drought decreased WHC both for DT cv. E70, especially DS cv. SY, but the application of *B. subtilis* 10-4 restored WHC indices to the control value, indicating that the bacterial priming protects plants from a drought-caused decrease in WHC indices. It should be noted that under normal conditions, *B. subtilis* 10-4 led to a significant increase in the WHC of leaves of cv. E70 (Figure 5A), while they had no effect on the WHC of Figure 5B, which indicates that DT cv. E70 is more responsive to *B. subtilis* 10-4 inoculation. The detected *Bacillus*-induced increase in WHC might be associated with their participation in the regulation of stomatal closure mechanisms, which helps plants minimize direct and indirect water consumption [62,63]. There is some evidence that *B. subtilis* affects plant growth and tolerance through stomatal regulation [64] with the involvement of ABA, which, in turn, is controlled by SA. Earlier, we revealed that inoculation with *B. subtilis* 10-4 led to the accumulation of endogenous SA in wheat seedlings under normal and salinity conditions [10]. It has been suggested that *B. subtilis* may activate the defense responses of wheat plants through the SA-dependent signaling pathways. With that, it is obvious that different cultivars may have different abilities to cope with environmental stresses, which may be due to both selection peculiarities in different ecological and geographical areas and an imbalance in hormone composition under the influence of *B. subtilis* [1,17,18].

SA or JA-dependent defense responses are the dominant primary signals of the local and systemic induced plant defense responses to stress [1,16], and the anti-stress effect of PGPB on plants may be related to their ability to synthesize these compounds [1,14,37,65,66]. SA participates in the regulation of various physiological processes including flowering, thermogenesis, stomatal closure, and ion transport as well as in the initiation of defense responses of plants against different biotic and abiotic stresses [67]. Previous studies have demonstrated that SA is a key phytohormone that plays a vital role in the growth and developmental process of plants and is actively involved in the response to drought stress through the activation of antioxidant enzymes, gene expression regulation, osmolyte synthesis (including Pro), and enhancement of photosynthetic pigment accumulation [67,68]. There is evidence that plant signaling pathways regulating the development of protective responses to stresses are the key targets of PGPBs [1,13]. For example, *B. subtilis* UMAF6639 formed established resistance to powdery mildew in melon through the activation of JA and SA-dependent defense reactions [14]. *B. cereus* AR156 induced systemic resistance of *Arabidopsis thaliana* via NPR-1 and the SA-dependent signaling pathway without affecting the JA/ET-dependent pathways [13]. The formation of *Pseudomonas*-primed chickpea resistance also occurred due to the production of phenolic compounds and the induction of systemic resistance through the SA-dependent signaling pathway [69]. Endophytic bacteria *Achromobacter xylosoxidans* and *B. pumilus* enhanced the growth of sunflower under water stress through endogenous SA production [14,70]. In wild-type tobacco plants, *B. amyloliquefaciens* FZB42 inoculation led to overexpression of the *PR-1a*, *LOX*, and *ERF1* genes involved in SA, JA, and ET-dependent signaling pathways [71]. With that, it remains unclear exactly how *B. subtilis* regulates the defense system of host plants under drought, and how the bacterial-induced plant defense system interacts with classical signaling pathways. It is believed that PGPB influences are similar to the effect of “weak” pathogens on plants, however, PGPB also may produce metabolites with hormonal and signaling functions (auxins, cytokinins, ethylene, gibberellins, abscisic acid, SA, JA, and others) [1,16]. Our results showed that under normal and drought stress conditions, the content of endogenous SA in wheat plants significantly varied depending on the cultivar and their susceptibility to water stress (Figure 8). In particular, DT cv. E70 wheat seedlings were characterized by much higher endogenous SA content than that of DS cv. SY (Figure 8). Given the DT behavior of cv. E70, it may be suggested that high endogenous SA level is likely to be one of the mechanisms allowing these plants to initiate defense mechanisms immediately when stressful situations such as drought occur, without inhibiting the growth. In contrast, under adverse conditions, DS cv. SY plants with a low level of SA initially begin to slow down their growth processes (Figure 3) and only afterwards accumulate compounds necessary to induce protective mechanisms. Interestingly, the observed difference between cultivars was maintained in their response to inoculation with *B. subtilis* (Figure 8). Rapid and high SA accumulation (with a peak in 4-old day seedlings) for cv. E70 was revealed while the increase in SA content was observed in five to six day old cv. SY seedlings. Under drought stress, there was a significant rise in endogenous SA content both for DT cv. E70 and DS cv. SY with a more pronounced SA increase in the DT cultivar. This phenomenon is consistent with the literature, as SA is a key mediator of signaling pathways leading to the activation of the plants’ defense systems in response to various stresses including drought [72,73]. Priming with *B. subtilis* 10-4 resulted in a decrease (up to 50%) in drought-caused endogenous SA accumulation depending on the cultivar and time of stress exposure (Figure 8C,D). These findings correspond with our data on the effect of *B. subtilis* 10-4 on plant growth under drought stress (Figure 3), which likely indicates the important role of endogenous SA in a *B. subtilis*-mediated protective effect on wheat plants of studied DT and DS cultivars. The observed similarities and differences of the *B. subtilis* 10-4 effects on endogenous SA in DT cv. E70 and DS cv. SY (differing by the ability to cope with drought stress) and their comparison with bacteria-induced changes in various indices (growth, WHC, photosynthetic pigments, and membrane damages) under normal and drought conditions suggest that *B. subtilis* 10-4 can induce defense responses through SA-dependent signaling pathways. The lower response of DS cv. SY to *B. subtilis* 10-4 inoculation might be connected with the involvement of other signaling protection pathways and the synthesis of other protective compounds, which may be incompatible with SA-dependent pathways (which inevitably leads either to the neutralization of the positive influence of *B. subtilis*, or to the inhibition of growth and development), or related to cv. The SY strategy is to hold back the growth processes at the beginning of the growing season, therefore it is advisable to carry out the treatment with growth-promoting bacterial preparations not only before sowing, but also during the growing season in the later stages of ontogenesis. These assumptions certainly need further detailed research.

## 5. Conclusions

Overall, we demonstrated that the endophytic bacteria *B. subtilis* 10-4 influences growth and physio-biochemical responses of DT and DS wheat cultivars E70 and SY in the early stages of ontogenesis. *B. subtilis* 10-4 significantly increased seed germination, elongation of seedlings, and biomass accumulation for DT wheat cv. E70 both under normal and drought conditions, but for DS cv. SY, there was no visible protective effect on growth under drought. *B. subtilis* 10-4 elevated levels of photosynthetic pigments Chl a, Chl b, and Car in leaves of cv. E70 both under normal and especially drought conditions, while cv. SY bacterial inoculation decreased Chl a, Chl b, and Car contents under normal conditions and almost recovered under drought stress. Additionally, *B. subtilis* 10-4 prevented the drought-induced decline in WHC in both cultivars. Notably, *B. subtilis* 10-4 increased endogenous SA level in both cultivars (especially in cv. E70) and reduced drought-induced endogenous SA accumulation, which was correlated with the influence of *B. subtilis* 10-4 on growth, indicating a possible involvement of endogenous SA in the implementation of *B. subtilis*-mediated effects on both cultivars. *B. subtilis* 10-4 inoculation was found to increase drought tolerance in seedlings of both cultivars evidenced by the decreased lipid peroxidation, Pro content, and EL from tissues of bacterial primed wheat seedlings under drought stress. The findings indicate *B. subtilis* 10-4 has the potential to be used as an eco-friendly agent to improve wheat growth and drought tolerance.
